# Anatomy of a famine: using fuzzy cognitive mapping to understand the crisis in Ayod County, South Sudan, 2017

**DOI:** 10.1057/s41271-026-00643-8

**Published:** 2026-08-12

**Authors:** Matthew Day

**Affiliations:** Independent Researcher, Hazard, KY USA

**Keywords:** Famine analysis, Complex systems, Fuzzy cognitive mapping, Humanitarian response

## Abstract

This paper explores Fuzzy Cognitive Mapping (FCM) as an analytical approach for understanding famine as a complex, dynamic system. Traditional approaches, such as the Integrated Food Security Phase Classification (IPC), may not fully capture the non-linear interactions among social, economic, political, and environmental factors that contribute to famine. The IPC relies heavily on cross-sectional survey data and expert judgment, which can introduce errors and biases. This study applies FCM to a retrospective case study of the 2017 Ayod County crisis in South Sudan. FCM combines qualitative and quantitative data, facilitating analysis of dynamic systems even with limited data availability. The findings indicate that FCM identified famine-like conditions from March to June 2017, aligning with subsequent survey data, although Ayod was never formally classified in famine by the IPC. Continued testing of FCM is recommended to assess its suitability as part of a revised analytical toolkit for near-real-time famine risk tracking.

**Supplementary Information:**

The online version contains supplementary material available at 10.1057/s41271-026-00643-8.

## Key messages

- Traditional famine tools like IPC may miss complex interactions driving famine. Fuzzy Cognitive Mapping (FCM) captures feedback loops and system dynamics that static threshold-based classifications cannot, potentially improving early warning.
- FCM applied to South Sudan’s 2017 Ayod crisis identified famine-like conditions months before formal assessments, demonstrating its potential as a complementary tool for near-real-time famine risk tracking in data-constrained environments.
- FCM could shift famine response from reactive to proactive by identifying intervention points and tracking system trajectories rather than waiting for threshold-based classifications that depend on large-scale survey data.

## Introduction

Famine can be conceptualized as an emergent state within a complex, dynamic system characterized by the interaction of social, economic, political, and environmental factors in non-linear patterns [1–5]. Howe [1] and Fortnam and Hailey [2] emphasize that famine dynamics exhibit cascading effects and self-reinforcing feedback loops that may propel populations toward a famine state, marked by starvation, acute malnutrition, disease outbreaks, and mass mortality [1, 2, 4–10].

Despite growing recognition of famine’s complexity, current analytical approaches lack systems-based methodologies and analytical tools [2–4]. The Integrated Food Security Phase Classification (IPC) relies on outcome severity indicators and static, threshold-based classification, potentially overlooking temporal, spatial, and processual dimensions of famine risk [1–9, 11, 12]. IPC Famine (Phase 5) classification requires randomly sampled survey evidence that outcome indicators exceed specific thresholds [13, 14], yet gathering reliable data for these measures is frequently challenging in conflict-affected contexts [3–5, 11, 15]. The IPC classification also involves multiple procedural stages, each adding complexity and potential for delay (see Supplement A for full IPC protocols, thresholds, and procedural detail).

The conditions signaling famine risk simultaneously degrade the evidence base. Conflict, restricted access, displacement, and isolation limit the feasibility of representative surveys precisely when evidence is most needed [1–5, 11, 12, 15, 16].

Quantitative models designed for predicting IPC Phase classifications, including famine, face critical limitations: IPC thresholds do not capture complex dynamic interactions, and classifications rely heavily on expert judgment rooted in imperfect information, increasing risk of errors, biases, and analytical noise [4, 9, 15, 17–20]. Such models essentially predict expert judgments rather than objectively measurable realities.

**Fig. 1:**
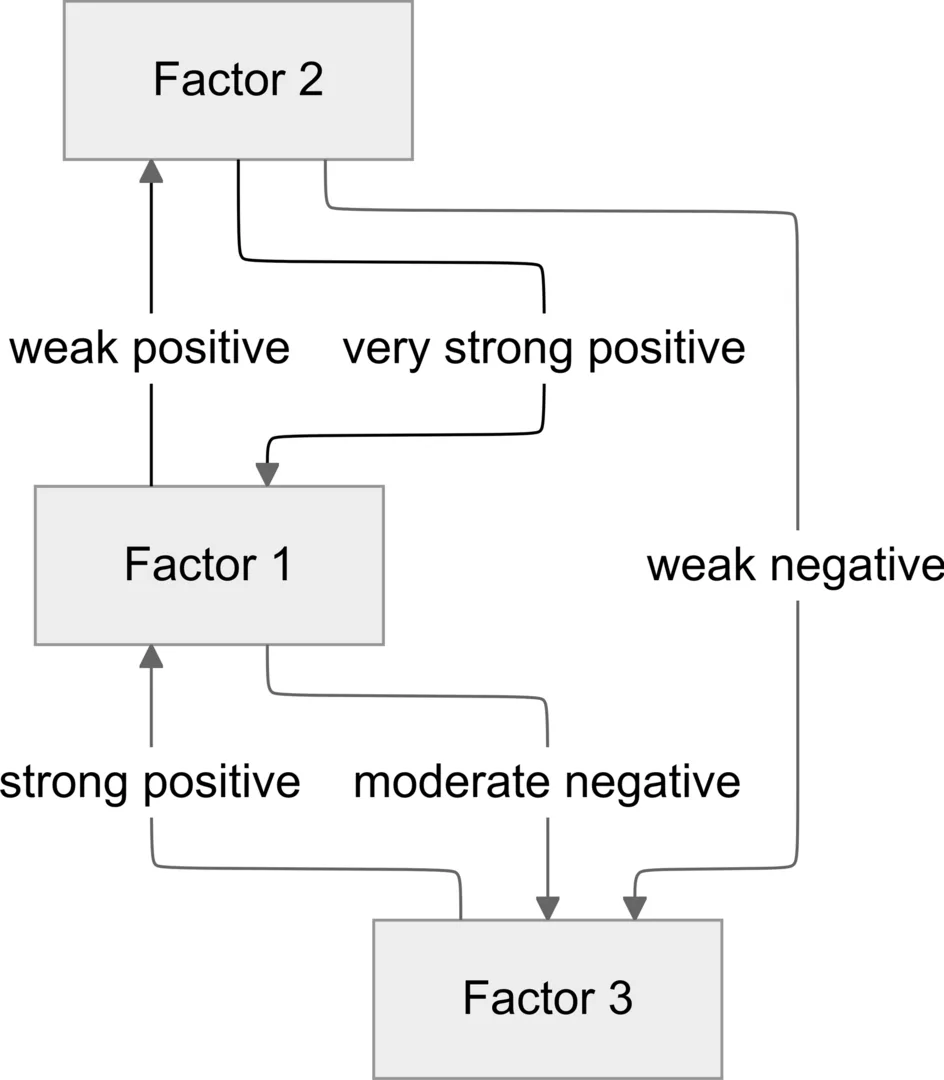
Example fuzzy cognitive map

Fuzzy Cognitive Mapping (FCM), developed by Kosko in 1986, is a practical method for modeling complex systems through weighted causal networks [21–28]. FCM’s critical strength lies in capturing feedback loops and emergent properties both visually and mathematically, enabling comprehensive analysis of dynamic systems [21–28]. FCM integrates diverse data types, supports scenario analysis, and generates system visualizations [23–25, 27, 28]. Despite widespread recognition that famine operates as a complex system, FCM has not been systematically evaluated for famine risk analysis. This paper applies FCM to a retrospective case study to assess its utility for tracking famine risk and identifying the critical pathways and feedback loops driving system deterioration toward famine (see Fig. 1).

Ayod County, South Sudan, situated within the Sudd in an agropastoral livelihood system, experienced severe flooding, disease outbreaks, and conflict between 2016 and 2017 [16, 29–37]. By early 2017, starvation and livelihood collapse led to near-complete reliance on wild foods, disease outbreaks, and widespread malnutrition, resulting in an IPC Acute Malnutrition (AMN) Phase 5 classification in May 2017 [38, 39]. A household survey in June 2017 reported 29% of households at IPC AFI Phase 5 Catastrophe [39], and the non-trauma crude death rate reached 1.89 (95%, CI: 1.26–2.84) per 10,000 per day—slightly below the famine threshold of 2.0 [40]. Local populations referred to it as “The Year of the Thou,” signifying complete dependence on tree leaves for sustenance [39]. Despite these indicators, Ayod was not classified as a famine by the IPC [41].

## Data and methods

This paper applies FCM to evaluate system states in Ayod from July 2016 to September 2017 through two complementary approaches. First, a static baseline FCM was constructed to identify contextually relevant factors and their causal relationships. Second, four dynamic FCM scenarios were developed by integrating time-bounded evidence and expert knowledge to model system trajectories across distinct crisis periods [22–28].

### Baseline FCM development

**Fig. 2:**
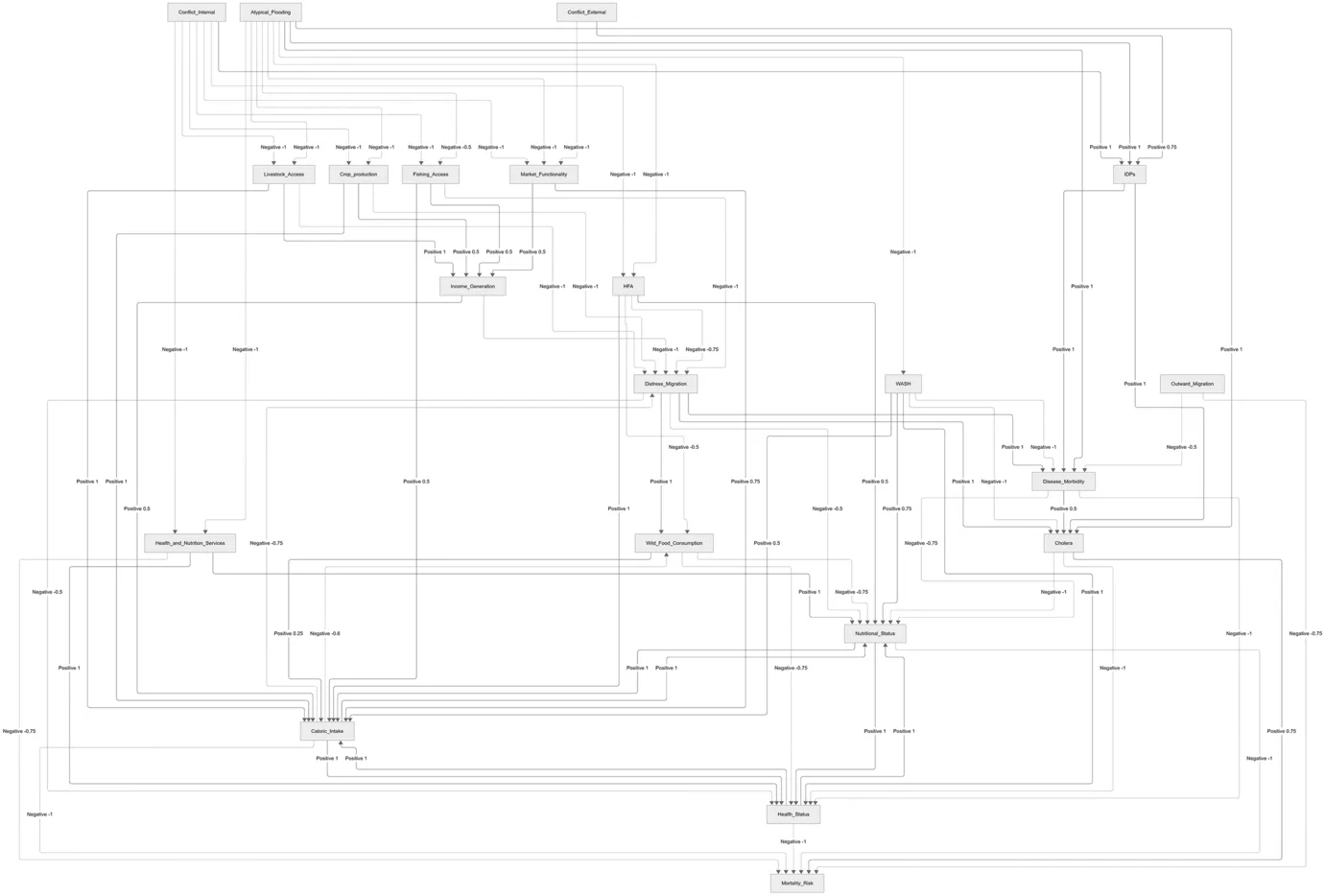
Ayod static (baseline) fuzzy cognitive map

The baseline FCM was constructed using the Mental Modeler software, selected for its user-friendly interface, low bandwidth demands, and collaborative advantages, which make it suitable for humanitarian contexts with diverse expertise and restricted technical capabilities [27, 28, 42, 43]. The model included 21 contextualized factors that directly or indirectly influence famine risk—caloric intake deficits, malnutrition prevalence, disease outbreaks, and non-trauma mortality rates [13, 14]. The concept of destitution—understood as the collapse of livelihood systems, household assets, and coping mechanisms—is not a distinct factor; rather, it is inferred through considerable reductions in livelihood-related factors. Factors were selected from diverse sources including the FEWS NET South Sudan livelihood zone report, gray literature, qualitative data from a rapid assessment conducted in Ayod in June 2017, and direct experience as a humanitarian analyst based in South Sudan from 2017–2020 [29, 39, 44, 45]. Relationships between factors were quantified by assigning weights to links within a range of – 1.0 to + 1.0 in 0.25 increments. A total of 74 causal relationships were defined, producing a model with a density of 0.176 and an average of 3.52 relationships per factor (see Supplement B for full factor list and descriptions; Supplement C for the baseline cognitive map) (see Fig. 2) (see Tables 1 and 2).

**Table 1:** Factors of interest for tracking risk of a famine state

Factor of interest	Description
Caloric Intake	Estimated food availability and consumption patterns, highlighting caloric deficits across different system states
Nutritional status	Assessed malnutrition prevalence, focusing particularly on vulnerable groups such as children under five
Health status	Tracked the potential for disease transmission, including cholera and acute watery diarrhea (AWD), alongside other health risks
Mortality risk	Evaluated the likelihood of increased mortality due to interactions between caloric deficets, malnutrition, and disease outbreaks

**Table 2:** 2016–2017 Ayod FCM factor list and descriptions

Factor	Description
Atypical flooding	Presence, extent, and impact of flooding that is not typical for the area, affecting various aspects of life and livelihoods
Livestock access	Ownership and ability to access livestock for food, income, and milk
Crop production	Seasonal crop production and its impact on food availability and income
Market functionality	Functionality of local markets, including the availability of goods and the ability to trade
Income generation	Income generated from selling own production, such as crops, livestock and fish, petty trade and labor
Fishing access	Ability to access and engage in fishing for food and income
WASH	General access to clean water, and sanitation and hygiene conditions in the area
Cholera	Cholera morbidity—proxy for likely cholera cases
IDPs	Number of internally displaced persons (IDPs) relative to the host population and their impact on resources and services
Caloric intake	The amount of calories consumed by an individual
Nutritional status	Overall nutritional status of the population, notably malnutrition prevalence among vulnerable groups
Disease morbidity	Prevalence of diseases such as malaria, acute watery diarrhea (AWD), or others
Health status	Health status of individuals, influenced by disease prevalence, nutrition, and access to healthcare
Conflict—internal	Presence of direct conflict within Ayod
Conflict—external	Presence of conflict in neighboring areas
HFA	Population’s access to humanitarian food assistance inflow to the area
Health and nutrition services	Availability and functionality of health and nutrition services in the area
Outward migration	Movement of people out of the area due to various factors such as conflict, lack of resources, or seeking better opportunities
Wild food consumption	Availability and frequency of wild food consumption as a supplement to regular food sources
Mortality risk	Risk of death due to various factors such as starvation, disease, malnutrition, and lack of healthcare
Distress migration	Internal atypical movement of people in search of resources and life-saving services

### Dynamic FCM analysis

Dynamic FCM analysis evaluated likely shifts in factor states relative to baseline by adjusting factor activation levels in response to distinct periods characterized by shifts in external pressures, seasonality, or significant events (see Supplement C, Table S7 for scenario descriptions). Activation values, ranging from –1 to + 1 in 0.25 increments, reflected relative changes in each factor using a convergence of qualitative and quantitative evidence [25–28]. Critically, factors such as caloric intake, nutritional status, health status, and mortality risk were not directly assigned values; instead, the model predicted their relative changes based on interrelationships with other factors, including feedback loops. Factors lacking sufficient evidence were treated similarly. Using the Mental Modeler software, the baseline FCM factor relationships and weights were applied to generate system state projections over time. Predictive performance was evaluated by comparing model-generated values of key indicators against retrospective data, contextual information, and observed outcomes. An evidence repository of 242 unique entries served as the foundational dataset for determining factor activation levels across scenarios (see Supplement C, Table S8 for full data sources) (see Table 3).

**Table 3:** Dynamic FCM system scenarios—time period and description

Dynamic FCM scenarios	Period	Typical livelihood season	Events
System state 1	July 2016–November 2016	Harvest period, livestock return to the homestead	Outbreak of national conflict and displacement into Ayod from Juba; atypical flooding in Ayod and additional internal displacement of populations in Ayod
System state 2	December 2016–February 2017	Dry season, some household food stocks remaining from harvest, livestock move away from the homestead	Outbreak of conflict in Ayod, with large advances into Ayod by SPLA, displacing populations in central Ayod to northern (Pagil) and western Ayod (low-lying Sudd)
System state 3	March to June 2017	Late dry period and early rainfall. Lean season, a traditional period when household consumption patterns decrease	Conflict reduces slightly in Ayod; SPLA makes large advances in Canal/Pigi and Fangak counties, north of Ayod; cholera outbreak in Ayod; collapse of northern trade corridor—a critical food and livestock supply route into Ayod
System state 4	July to September 2017	Early harvest, some green harvest available, livestock return to the homestead	Significant upscale in humanitarian food and nutrition distributions

## Results

The FCM analysis across four system states revealed a progressive deterioration of conditions in Ayod from July 2016 through June 2017, followed by initial recovery.

### System state 1 (July–November 2016)

**Fig. 3:**
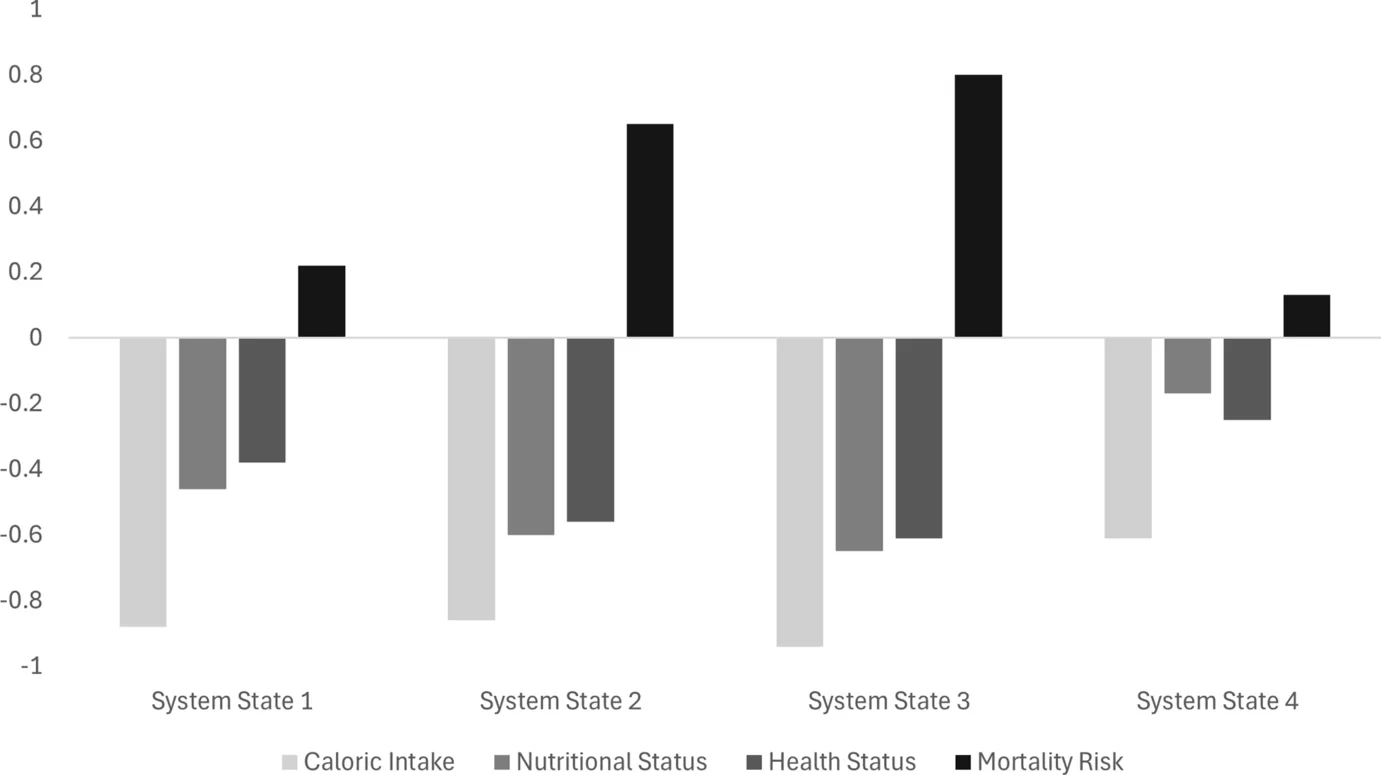
Dynamic FCM results 2016–2017

Severe flooding caused significant environmental and socio-economic disruptions. National conflict resulted in large influx of IDPs into Ayod. Livestock access declined sharply, crop production collapsed, and market functionality deteriorated, driving increased reliance on wild foods [44–46]. The FCM predicted substantial declines across all four focal factors (caloric intake, health status, nutrition status, and mortality risk), with caloric intake most severely affected (see Fig. 3).

### System state 2 (December 2016–February 2017)

Pressures intensified with escalating conflict and the early lean season. Markets remained non-functional, households exhausted food stocks, and first cholera cases emerged from poor WASH conditions and population concentration [31, 44, 45, 47]. The FCM indicated continued deterioration across all focal factors, with mortality risk rising sharply.

### System state 3 (March–June 2017)

The peak of the crisis. Prolonged food shortages, intensified disease outbreaks, and mass displacement converged to create famine conditions. Livelihood systems and markets collapsed, humanitarian assistance was severely constrained, and households relied almost entirely on nutritionally inadequate wild foods. The FCM predicted the most severe deterioration during this period, with near-total caloric depletion and significant mortality risk.

### System state 4 (July–September 2017)

Signs of recovery emerged with seasonal improvements, partial livelihood restoration, and expanded humanitarian interventions. Access to livestock improved, start of the harvest, markets showed modest recovery, and a cholera vaccination campaign reduced disease transmission. The FCM reflected marked improvement across all focal factors, though caloric intake remained well below baseline.

Figure 3 illustrates the changes in the four factors of interest across each scenario iteration, reflecting likely shifts during each system state identified (see Supplement C, Table S9 for the Dynamic FCM full activation and predicted values matrix).

## Discussion

### Alignment with survey data

**Fig. 4:**
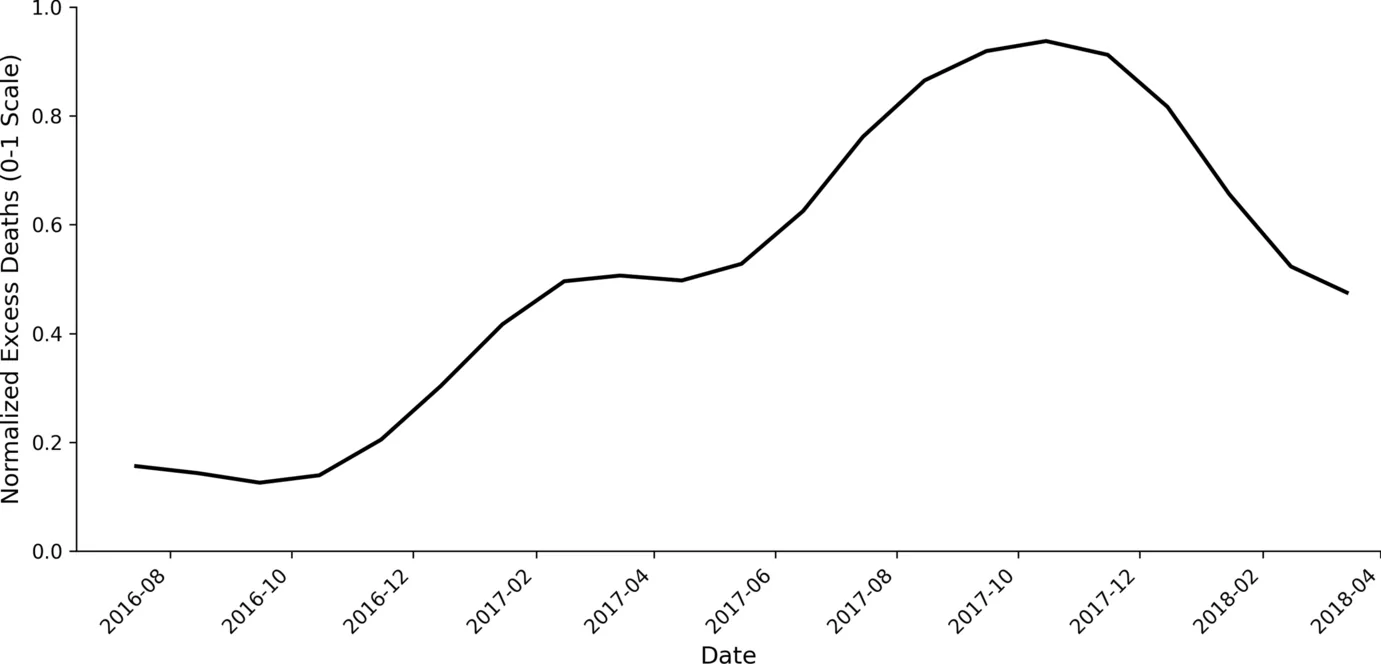
Normalized excess deaths in Ayod County, South Sudan (July 2016–September 2017) (Source: Francesco Checchi et al., 2018; adapted by Author)

The FCM indicated famine-like conditions from March to June 2017, aligning with the findings from a REACH household survey reporting 29% of households reporting very severe hunger via household hunger score (HHS), indicative of IPC Phase 5 AFI, in June 2017 [39]. FCM also indicated improvement by August–September 2017, consistent with FSNMS data showing 84% of households in Phase 3, according to the HHS (see Tables 4, 5, 6). The FCM effectively demonstrated shifts in malnutrition levels: GAM rates from multiple MUAC-based screenings (ranging from 26 to 34.2%) during System States 2–3 exceeded the IPC Phase 5 threshold of 15%, consistent with FCM conclusions, and aligned with decreased GAM prevalence captured through SMART survey in August–September 2017 (see Table 7). Comparison with time-bound normalized version of Checchi et al. [48] mortality model showed partial alignment, though Checchi’s model does not distinguish between trauma and non-trauma mortality, limiting direct comparability (see Fig. 4). Discrepancies in the timing of mortality reduction between System States 3 and 4 suggest areas for model refinement (see Supplement D for full survey tables, nutrition data, and mortality model comparison).

**Table 4:** REACH household survey results (June 2017)

Household hunger score	Percentage	n-count
Phase 3	41%	33
Phase 4	30%	24
Phase 5	29%	23

**Table 5:** FSNMS household survey results (August 2017)

Household hunger score	Percentage	n-count
IPC phase 1	12%	7
IPC phase 2	4%	2
IPC phase 3	84%	48

**Table 6:** SMART survey results (August–September 2017)

Household hunger score	Percentage	n-count
IPC phase 2	8.40%	36
IPC phase 3	82%	354
IPC phase 4	9.60%	41

**Table 7:** Nutrition screening and surveys—Ayod County, 2017

Date	Source	Methodology	Locations	Children Screened	GAM U5 (MUAC)	SAM U5 (MUAC)
Jan 12–16, 2017	Kandak RRM	Mass MUAC Screening	Kandak (Northern Ayod)	1200	34.2%	4.3%
Feb 1–4, 2017	WFP-CRS RNA	Ad-Hoc screening (non-random)	Normanyang, Karmoun, Buot, Wani	115	26.0%	N/A
Apr 21, 2017	Normanyang RRM	Mass MUAC screening	Normanyang (Northern Ayod)	1713	34.2%	8.17%
June 20–26, 2017	REACH household survey	Two-stage PPS random sampling	Pagil, Gorwai, Jeich, Yein, Haat	160	21.3%	7.5%
Aug 21–Sept 16, 2017	SMART survey	Two-stage cluster sampling	Mongok, Pajiek, Wau, Pagil Payams	596	13.4%	4.5%

### IPC methodological limitations

The September 2017 IPC assessment had access to three surveys, each with distinct limitations [41]. The REACH survey (June 2017) reported Phase 5-level food insecurity; the FSNMS (July–August 2017) had limited geographic scope; and the SMART survey (August–September 2017) was affected by response bias, with over 50% of clusters having received food assistance within two weeks prior to data collection. Notably, the initial non-trauma CDR from the SMART survey was 2.03—exceeding the famine threshold [49]—but was later reduced to 1.89 in the final report [50] through an extension of the recall period from 107 to 126 days. That three surveys conducted within months of each other produced conflicting assessments of the same crisis reflects not merely a data problem but a systemic limitation of snapshot-based assessment (see Supplement D for detailed survey critique).

### Value add of FCM

The baseline FCM enabled visualization and analysis of the complex interrelationships influencing Ayod’s trajectory toward famine, producing a contextualized system blueprint that static classification methods cannot replicate. The model captured tipping points where prolonged pressures and collapsing adaptive capacity accelerated self-reinforcing feedback loops—particularly during System State 3, when converging food shortages, cholera, and displacement drove the system toward collapse (see Supplemental Material D and E). It also identified how humanitarian food assistance acted as a rebalancing mechanism in System State 4, disrupting these feedback loops and steering the system away from famine. Critically, the FCM identified famine-like conditions from March to June 2017, the IPC classified Ayod’s acute malnutrition at Phase 5 in May 2017, and the IPC never declared famine despite indicators approaching or exceeding famine thresholds. This timing difference illustrates FCM’s potential as an early warning complement: by tracking system trajectories rather than waiting for threshold-based classification, FCM has the potential to signal emerging risk before formal declarations, and enable development of contextualized near-real-time famine risk tracking system. Furthermore, FCM is not subject to the methodological fragilities inherent in threshold-based systems. The SMART survey’s non-trauma CDR shifted from 2.03 to 1.89—crossing the famine threshold—solely because a 19-day recall period extension added no new deaths but diluted the rate. Such threshold sensitivity does not affect FCM, which tracks relative system trajectories rather than binary classification thresholds. FCM also supported identification of non-linear interactions and emergent properties—atypical flooding, conflict dynamics, immobility, and a cholera outbreak—that amplified each other and drove the system toward famine through self-reinforcing feedback loops. (see Supplement E for extended systems analysis).

### Limitations

There are multiple limitations to consider. This represents a single application of FCM to one famine context; broader conclusions require further testing across diverse settings. The Ayod FCM was developed using available evidence and expertise rather than through a participatory process involving a range of stakeholders. Validating FCM outputs against empirical outcomes remains difficult in humanitarian contexts with limited data. Future applications should prioritize participatory development and establish rigorous validation protocols (see Supplement E, Table S14 for a detailed assessment of FCM’s strengths and weaknesses in the Ayod case).

## Conclusions

Although this case study was conducted retrospectively, the FCM construction relied solely on data available between July 2016 and August 2017. This approach simulated a quasi-real-time analysis, reflecting how the system might have been understood if the model had been used during the crisis. While it remains uncertain whether such an approach would have influenced decision-making at the time, the exercise underscores FCM’s potential to support early identification of emerging risks. With adequate buy-in from decision-makers, FCM could serve as a dynamic framework for guiding targeted interventions and informing strategic responses in future crises. Rather than positioning FCM as a replacement for the IPC, this study highlights its potential as a complementary analytical tool that addresses aspects of famine analysis that the IPC framework was not designed to capture.

Future efforts should explore FCM as a decision support tool for famine analysts, with a focus on the following:

- **Stakeholder integration:** Engaging humanitarian organizations, decision-makers, and local populations in the FCM development and analysis process.
- **Tool integration:** Combining FCM with complementary tools, such as GIS analysis, competing hypothesis analysis, and other standard analytical techniques to mitigate expert judgment errors in data-limited settings.
- **Near-real-time application:** Utilizing FCM for real-time system analysis to improve early warning systems, enable timely interventions, and support effective famine prevention strategies.

This case study demonstrates the potential value of adopting a dynamic, systems-based perspective through FCM, in which analysts can gain a more comprehensive understanding of famine trajectories and support more effective humanitarian decision-making, shifting famine response from reactive to proactive.

## Supplementary Information

Below is the link to the electronic supplementary material.

Supplementary material 1 (DOCX 630 kb)

Supplementary material 2 (XLSX 38 kb)

Supplementary material 3 (MMP 52 kb)

## Data Availability

Data is provided within the manuscript or supplementary information files—including the mentalmodel project file (.mmp).
