# Supplementary material for: Anatomy of a famine: using fuzzy cognitive mapping to understand the crisis in Ayod County, South Sudan, 2017
Source: J Public Health Policy. 2026 Aug 12;47(Suppl 1):56–70. doi: 10.1057/s41271-026-00643-8 (PMC13503268; doi:10.1057/s41271-026-00643-8)
Supplement: Supplementary file 1 [file 41271_2026_643_MOESM1_ESM.docx]

**Supplementary Material**

Anatomy of a famine: using fuzzy cognitive mapping to understand the crisis in Ayod County, South Sudan, 2017

Matthew Day

BSc (Economics, Louisiana State University), MSc (Developmental Practice, Trinity College Dublin)

Independent Researcher, Hazard, KY, USA

**Supplement A: IPC Famine Classification Framework**

This supplement provides detailed information on the Integrated Food Security Phase Classification (IPC) framework for famine classification, including its protocols, thresholds, and procedural complexities, as well as a direct comparison with the Fuzzy Cognitive Mapping approach used in the main paper.

**IPC Famine (Phase 5) Classification Thresholds**

According to IPC guidelines, classifying a Famine (IPC Phase 5) requires that two of the following three indicators exceed specific thresholds (13):

• At least 20% of households face extreme food deficit.

• Global Acute Malnutrition (GAM) prevalence ≥30% among children under five (≥15% if using MUAC).

• Non-trauma Crude Death Rate (CDR) ≥2 deaths per 10,000 people per day.

However, reliance on these static, threshold-based indicators has significant limitations, especially in conflict-affected or inaccessible contexts (3,4,11,12). Gathering reliable, representative, and timely data for these outcome measures is frequently challenging (4,11,12). Cross-sectional surveys, which dominate IPC assessments, offer limited insights into how crises evolve dynamically, potentially hindering prompt identification and response (3,12).

**IPC Classification Protocols and Procedural Complexity**

The IPC classification involves multiple procedural stages, each adding complexity and requiring substantial coordination, potentially introducing delays and susceptibility to political influence:

**Table S1: Overview of IPC Protocols for Famine Classification (14).**

| **Stage** | **Description** |
| --- | --- |
| **Technical Consensus** | Requires extensive multidisciplinary consultation, potentially delaying rapid consensus formation in rapidly changing environments. |
| **Evidence Collection** | Emphasis on “robust” direct evidence (R2) or complicated inference methods introduces potential subjectivity and biases, particularly under data scarcity. |
| **Unit of Analysis** | Population size minimum (10,000 people) can obscure localized famine conditions, particularly in smaller, isolated communities. |
| **Methodological Standards** | Strict requirements for methodological rigor (≥150 cases or ≥25 clusters) and temporal alignment can limit flexibility, affecting responsiveness during rapidly evolving crises. Reliance on livelihood baselines up to 10 years old might inadequately reflect recent livelihood disruptions. |
| **Quality Assurance** | A two-tier review, while intended to mitigate bias, can introduce procedural delays, possibly postponing critical humanitarian responses. |
| **Communication** | Formal alerts require meticulous documentation and clarity, further lengthening the time between crisis onset and recognition. |

These methodological and procedural complexities—combined with the IPC’s inherent reliance on static indicators—risk delaying timely famine classification and exacerbate humanitarian consequences. For detailed IPC Famine protocols, refer to IPC Special Protocols (14).

**Limitations in Quantitative Modeling of Famine**

Recent advances in systems thinking, artificial intelligence, and computational modeling offer promising tools for analyzing famine dynamics (12,15,48,52–56). However, quantitative models designed around predicting IPC Phase classifications, particularly Phase 5 (Famine), face critical limitations.

Firstly, IPC thresholds do not effectively capture the complex, dynamic interactions among factors such as livelihood collapse, market disruptions, water sanitation and hygiene (WASH), environmental shocks, conflict, and disease outbreaks, all of which influence famine outcomes (1,2,4,15,57). Secondly, IPC classifications heavily rely on expert judgment, inherently prone to errors, biases, and noise arising from incomplete or imperfect information (11,17–20). Thus, models anchored to IPC outcomes essentially predict expert judgments rather than objectively measurable realities like caloric deficits, acute malnutrition, or non-trauma mortality. Consequently, such quantitative models struggle to represent the full complexity of famine systems, which include non-linear dynamics, feedback loops, and critical tipping points (1,2,4).

**Comparing IPC and FCM Approaches**

Table S2 summarizes key methodological differences between the IPC and the modified FCM approach applied in this study. These differences are presented to contextualize the FCM application described in the main paper, not to evaluate one approach against the other.

**Table S2: Summary of Methodological Differences Between IPC and FCM**

| **Feature** | **IPC** | **FCM (as applied)** |
| --- | --- | --- |
| **Primary objective** | Classification of acute food security and malnutrition status according to standardized phases (1–3,6,7) using evidence-based thresholds (13,14) | Tracking dynamic shifts in factors related to famine risk without assigning a formal classification |
| **Data requirements** | Evidence must meet specific reliability criteria (R1/R2) with minimum requirements per evidence level (13) | Incorporates diverse data sources including quantitative data, qualitative assessments, and expert judgments |
| **Treatment of qualitative data** | Used primarily to contextualize quantitative findings and support convergence of evidence (13) | Directly integrated into the model as factor values and relationship weights |
| **Temporal dynamics** | Classification periods with current and projected phases; projections based on most likely scenario assumptions (14) | Dynamic modeling of system evolution over time, incorporating feedback loops |

**References**

1. Howe P. Famine systems: A new model for understanding the development of famines. World Development. 2018 May;105:144–55.

2. Fortnam M, Hailey P. Insights from social-ecological systems thinking for understanding and preventing famine. Disasters. 2024 Jul;48(3):e12621.

3. Maxwell D, Hailey P. Towards Anticipatory Information Systems and Action: Notes on Early Warning and Early Action in East Africa. Boston: Feinstein International Center, Tufts University; 2020.

4. Maxwell D, Khalif A, Hailey P, Checchi F. Viewpoint: Determining famine: Multi-dimensional analysis for the twenty-first century. Food Policy. 2020 Apr;92:101832.

11. Maxwell D, Hailey P. The Politics of Information and Analysis in Famines and Extreme Emergencies: Synthesis of Findings from Six Case Studies. Boston: Feinstein International Center, Tufts University; 2020.

12. Lentz E, Gottlieb G, Simmons C, Maxwell D. 2020 Hindsight? The Ecosystem of Humanitarian Diagnostics and Its Application to Anticipatory Action. Boston: Feinstein International Center, Tufts University; 2020.

13. IPC Global Partners. Integrated Food Security Phase Classification Technical Manual Version 3.1. Rome; 2021.

14. Integrated Food Security Phase Classification (IPC). IPC Famine Classification – Special Additional Protocols. 2021.

15. Howe PW, Naumova EN. Poverty and famines 2.0: the opportunities and challenges of crisis modeling and forecasting. J Public Health Pol. 2022 Sep;43(3):329–34.

48. Checchi F, Testa A, Warsame A, Quach L, Burns R. Estimates of crisis-attributable mortality in South Sudan, December 2013–April 2018. London School of Hygiene and Tropical Medicine; 2018.

17. Kahneman D, Sibony O, Sunstein CR. Noise: a flaw in human judgment. Little, Brown Spark; 2022.

18. Tversky A, Kahneman D. Judgment under Uncertainty: Heuristics and Biases. Science. 1974;185(4157):1124–31.

19. Korteling JE, Paradies GL, Sassen-van Meer JP. Cognitive bias and how to improve sustainable decision making. Front Psychol. 2023;14:1129835.

20. Heuer RJ. Psychology of intelligence analysis. Washington, D.C.: CIA; 1999.

52. Way-Henthorne J. Why tailored forecasts work so well for the eastern Horn of Africa March-to-May rainy season. Climate Hazards Center. 2022.

53. Engler S. Developing a historically based “Famine Vulnerability Analysis Model” (FVAM). ERDKUNDE. 2012 Jun;66(2):157–72.

54. Checchi F, Testa A, Gimma A, Koum-Besson E, Warsame A. A method for small-area estimation of population mortality in settings affected by crises. Popul Health Metrics. 2022;20(1):4.

55. Carneiro B, Perfetto C, Resce G, Ruscica G, Tucci G. Food insecurity trends in the Famine Early Warning Systems Network. In: CARMA 2023. 2023.

56. Kazemi Garajeh M, et al. An integrated approach of remote sensing and geospatial analysis for modeling and predicting the impacts of climate change on food security. Scientific Reports. 2023.

57. Righettini MS, Bordin E. Exploring food security as a multidimensional topic. Qual Quant. 2023;57(3):2739–58.

**Supplement B: Fuzzy Cognitive Mapping — Methodology and Construction**

This supplement details the Fuzzy Cognitive Mapping (FCM) methodology applied in the main paper, including its theoretical foundations, construction steps, factor definitions, and the activation scale used for dynamic scenario analysis.

**Theoretical Foundations**

Building on Howe’s (1) conceptualization of famine as a complex, dynamic system and Fortnam and Hailey’s (2) framing of famine as a social-ecological system (SES), the main paper tests a modified use of Fuzzy Cognitive Mapping (FCM) to track a system’s trajectory towards a famine state. Howe emphasizes that famines emerge from “synergistic interactions” among factors, forming self-reinforcing dynamics that push a system towards famine (1). Similarly, Fortnam and Hailey argue that a linear perspective fails to capture the non-linear interactions and feedback loops in famine systems (2).

FCM, developed by Kosko in 1986, offers a practical method for operationalizing these theoretical approaches (21–24). FCM is a method used to model complex systems by representing the relationships between different factors as a network of nodes and edges. Each node represents a concept or factor, and each edge represents the influence one factor has on another. The strength of these influences is expressed using fuzzy logic, which allows for degrees of influence rather than binary relationships. FCM is particularly useful in fields like environmental management, social sciences, and decision-making processes where systems are complex and data may be uncertain or incomplete (see Figure S1) (22–27,58–64). Unlike traditional models, FCMs capture feedback loops and emergent properties, potentially providing critical insights for understanding of famine systems.

**Table S3: Core Strengths of FCM**

| **Core Strengths of FCM** | **Description** |
| --- | --- |
| **Data Integration** | Combines qualitative and quantitative data for system representation, aligning with SES principles. |
| **Stakeholder Engagement** | Iterative development with expert input to identify factors and causal relationships. |
| **Dynamic Analysis** | Supports real-time scenario analysis to monitor system shifts and detect early warning signs. |
| **Visualization** | Generates graphical representations to clarify system dynamics, interactions, and feedback loops. |

**Key Steps in FCM Construction**

Key steps in FCM construction include the following (21,24,25,61):

• **Establishing Factors:** Identify and define key system variables (e.g., conflict, mobility, market prices, rainfall, disease prevalence, water availability).

• **Mapping Causal Relationships:** Determine how factors influence one another, capturing the complexity of interactions and potential tipping points.

• **Assigning Weights:** Determine the strength and direction of the influence between factors, usually using values ranging from –1 to +1. These fuzzy weights represent the degree to which one factor influences another. Positive values indicate a reinforcing relationship, while negative values represent an inhibiting effect.

• **Building the FCM:** Construct a directed graph representing factors and weighted causal links, offering a clear visual of system behavior.

• **Analyzing the FCM:** Perform static and dynamic analysis to observe how factor changes propagate through the system, identifying vulnerabilities and early warning signals.

*Figure S1: Example Fuzzy Cognitive Map*

**FCM Tool Selection and Workflow**

The FCM for Ayod was created through a two-step process: initially establishing a baseline map, followed by conducting four “what-if” scenario analyses using the Dynamic FCM methodology (25,28,62). The author employed Mental Modeler, an online tool selected for its user-friendly interface, low bandwidth demands, and collaborative advantages, which make it suitable for humanitarian contexts with diverse expertise and restricted technical capabilities (23,42,43). While it lacks advanced machine learning functions, it facilitates baseline mapping and straightforward scenario analysis, aiding stakeholders in comprehending complex systems (23,42).

*Figure S2: Methodology Workflow — FCM Baseline and Scenario Analysis*

**Factors of Interest for Tracking Risk of a Famine State**

**Table S4: Factors of Interest for Tracking Risk of a Famine State**

| **Factor of Interest** | **Description** |
| --- | --- |
| **Caloric Intake** | Estimated food availability and consumption patterns, highlighting caloric deficits across different system states. |
| **Nutritional Status** | Assessed malnutrition prevalence, focusing particularly on vulnerable groups such as children under five. |
| **Health Status** | Tracked the potential for disease transmission, including cholera and acute watery diarrhea (AWD), alongside other health risks. |
| **Mortality Risk** | Evaluated the likelihood of increased mortality due to interactions between food insecurity, malnutrition, and disease outbreaks. |

**Baseline FCM Factor Definitions**

The FCM approach developed by the author corresponds to famine outcome criteria, emphasizing four key factors: caloric intake deficits, malnutrition prevalence, disease outbreaks, and non-trauma mortality rates (13,14). The concept of destitution—understood as the collapse of livelihood systems, household assets, and coping mechanisms—is not separately included but inferred through considerable reductions in aspects such as a household’s asset base and coping capacity. Factors for the model were selected based on diverse sources, including the FEWS NET South Sudan livelihood zone report, grey literature, qualitative data from a rapid assessment conducted by the author in June 2017, and the author’s experience as a humanitarian analyst based in South Sudan from 2017–2020. The baseline FCM included 21 factors, which were also integrated into the dynamic FCM scenario analysis.

**Table S5: 2016–2017 Ayod FCM Factor List and Descriptions**

| **Factor** | **Description** |
| --- | --- |
| **Atypical Flooding** | Presence, extent, and impact of flooding that is not typical for the area, affecting various aspects of life and livelihoods. |
| **Livestock Access** | Ownership and ability to access livestock for food, income, and milk. |
| **Crop Production** | Seasonal crop production and its impact on food availability and income. |
| **Market Functionality** | Functionality of local markets, including the availability of goods and the ability to trade. |
| **Income Generation** | Income generated from selling own production, such as crops, livestock and fish, petty trade and labor. |
| **Fishing Access** | Ability to access and engage in fishing for food and income. |
| **WASH** | General access to clean water, and sanitation and hygiene conditions in the area. |
| **Cholera** | Cholera morbidity – proxy for likely cholera cases. |
| **IDPs** | Number of internally displaced persons (IDPs) relative to the host population and their impact on resources and services. |
| **Caloric Intake** | The amount of calories consumed by an individual. |
| **Nutritional Status** | Overall nutritional status of the population, notably malnutrition prevalence among vulnerable groups. |
| **Disease Morbidity** | Prevalence of diseases such as malaria, acute watery diarrhea (AWD), or others. |
| **Health Status** | Health status of individuals, influenced by disease prevalence, nutrition, and access to healthcare. |
| **Conflict – Internal** | Presence of direct conflict within Ayod. |
| **Conflict – External** | Presence of conflict in neighboring areas. |
| **HFA** | Population’s access to humanitarian food assistance inflow to the area. |
| **Health and Nutrition Services** | Availability and functionality of health and nutrition services in the area. |
| **Outward Migration** | Movement of people out of the area due to various factors such as conflict, lack of resources, or seeking better opportunities. |
| **Wild Food Consumption** | Availability and frequency of wild food consumption as a supplement to regular food sources. |
| **Mortality Risk** | Risk of death due to various factors such as starvation, disease, malnutrition, and lack of healthcare. |
| **Distress Migration** | Internal atypical movement of people in search of resources and life-saving services. |

**Weight Assignment Methodology**

Relationships between factors were quantified by assigning weights to links within a range of –1.0 to +1.0 in 0.25 increments. Positive weights indicate a direct relationship (e.g., an increase in one factor results in an increase in another), whereas negative weights signify inverse relationships. The magnitude reflects the strength of the relationship, with larger absolute values denoting a stronger influence (21,24,25). A total of 74 causal relationships were defined, producing a model with a density of 0.176 and an average of 3.52 relationships per factor. The FCM’s complexity score, which evaluates the balance among drivers, receivers, and intermediate factors, was 0.25.

**Table S6: Dynamic FCM Factor Activation Scale**

| **Factor Change during Scenario Period** | **Value** |
| --- | --- |
| Significantly increase | 1 |
| Increase | 0.75 |
| Moderate increase | 0.5 |
| Slight increase | 0.25 |
| Slight decrease | –0.25 |
| Moderate decrease | –0.5 |
| Decrease | –0.75 |
| Significant decrease | –1 |

**Dynamic FCM Methodology**

Dynamic FCM analysis applied scenario-based system modeling by adjusting factor activation levels in response to distinct periods characterized by shifts in external pressures, seasonality, or significant events. Activation values, ranging from –1 to 1 in 0.25 increments, reflected relative changes in each factor using a convergence of qualitative and quantitative evidence (25–28).

The author did not assign activation values directly to the four focal factors—caloric intake, nutritional status, health status, and non-trauma mortality risk. Instead, the fuzzy cognitive map (FCM) estimated their relative changes across system states based on dynamic interrelationships and feedback loops with other variables. To reduce outcome bias, the author deliberately excluded direct outcome evidence—such as caloric intake, livelihood change, malnutrition prevalence, and non-trauma mortality—when setting activation values across scenarios.

Apart from the four focal factors, scenario-specific activation values for all other variables were derived through expert judgment, drawing on the complete body of indirect evidence available during the relevant scenario period or from preceding periods. Direct outcome evidence refers to household survey data used by the Integrated Food Security Phase Classification (IPC) to assess acute food insecurity outcomes and famine classification (13,14). These include the Household Hunger Scale (HHS), Food Consumption Score (FCS), Livelihood Coping Strategies (LCS) module, Reduced Coping Strategy Index (RCSI), global acute malnutrition (GAM) prevalence by weight-for-height, proxy GAM by mid-upper arm circumference (MUAC), and crude mortality rates (CDR) excluding trauma-related deaths (13).

The term “proxy GAM by MUAC” includes both survey-based MUAC data from randomized household sampling and MUAC data collected through mass screenings during food distributions or clinic-based nutrition screenings. However, per IPC protocols, MUAC data from individual site screenings or admissions at nutrition clinics are not considered valid direct evidence for malnutrition outcome classification (13,14).

Using the Mental Modeler software, the baseline FCM factor relationships and weights were applied to generate system state projections over time. For each of the four system states (scenarios), activation levels were adjusted based on the qualitative and quantitative evidence from the corresponding period. The software then propagated these changes through the model, generating predicted shifts in caloric intake, nutritional status, health status, and mortality risk.

Although the author sought to minimize reliance on direct outcome evidence when determining factor activation values, retrospective bias remains a potential limitation—particularly for the four focal factors, which were modeled endogenously by the FCM. The FCM’s performance was evaluated by comparing its predicted shifts in caloric intake, nutritional status, health status, and non-trauma mortality risk for each system state (scenario) against both retrospective analyses, and direct outcome data collected through household surveys during the scenario period.

**References**

1. Howe P. Famine systems: A new model for understanding the development of famines. World Development. 2018 May;105:144–55.

2. Fortnam M, Hailey P. Insights from social-ecological systems thinking for understanding and preventing famine. Disasters. 2024 Jul;48(3):e12621.

13. IPC Global Partners. Integrated Food Security Phase Classification Technical Manual Version 3.1. Rome; 2021.

14. Integrated Food Security Phase Classification (IPC). IPC Famine Classification – Special Additional Protocols. 2021.

21. Kosko B. Fuzzy cognitive maps. International Journal of Man-Machine Studies. 1986 Jan;24(1):65–75.

22. Aravindakshan S, et al. Socio-cognitive constraints and opportunities for sustainable intensification in South Asia. Environ Dev Sustain. 2021;23(12):16588–616.

23. Blacketer MP, Brownlee MTJ, Baldwin ED, Bowen BB. Fuzzy Cognitive Maps of Social-Ecological Complexity: Applying Mental Modeler to the Bonneville Salt Flats. Ecological Complexity. 2021 Sep;47:100950.

24. Gray SA, et al. Using fuzzy cognitive mapping as a participatory approach to analyze change, preferred states, and perceived resilience of social-ecological systems. E&S. 2015;20(2):art11.

25. Barbrook-Johnson P, Penn AS. Participatory Systems Mapping. In: Systems Mapping. Springer; 2022. p. 61–78.

26. Ozesmi U, Ozesmi SL. Ecological models based on people’s knowledge: a multi-step fuzzy cognitive mapping approach. Ecological Modelling. 2004 Aug;176(1,2):43–64.

27. Papageorgiou EI, editor. Fuzzy Cognitive Maps for Applied Sciences and Engineering. Berlin: Springer; 2014.

28. Felix G, et al. A review on methods and software for fuzzy cognitive maps. Artif Intell Rev. 2019;52(3):1707–37.

42. Gray SA, Gray S, Cox LJ, Henly-Shepard S. Mental Modeler: A Fuzzy-Logic Cognitive Mapping Modeling Tool for Adaptive Environmental Management. In: Proceedings of the 46th Hawaii International Conference on System Sciences. IEEE; 2013. p. 965–73.

43. Mental Modeler [Internet]. [cited 2024]. Available from: https://www.mentalmodeler.com/

58. Ghaboulian Zare S, et al. Examining wind energy deployment pathways in complex macro-economic and political settings using a fuzzy cognitive map-based method. Energy. 2022 Jan;238:121673.

59. Khodadadi M, et al. Fuzzy cognitive map based approach for determining the risk of ischemic stroke. IET Syst Biol. 2019 Dec;13(8):297–304.

60. Large Scale Systems and Fuzzy Cognitive Maps: A critical overview of challenges and research opportunities.

61. Van Vliet M, Kok K, Veldkamp T. Linking stakeholders and modellers in scenario studies: The use of Fuzzy Cognitive Maps as a communication and learning tool. Futures. 2010 Feb;42(1):1–14.

62. Papageorgiou EI, Salmeron JL. A Review of Fuzzy Cognitive Maps Research During the Last Decade. IEEE Trans Fuzzy Syst. 2013 Feb;21(1):66–79.

63. Papageorgiou E, Kontogianni A. Using Fuzzy Cognitive Mapping in Environmental Decision Making and Management. InTech; 2012.

64. Mourhir A. Scoping review of the potentials of fuzzy cognitive maps as a modeling approach for integrated environmental assessment and management. Environmental Modelling & Software. 2021 Jan;135:104891.

**Supplement C: Ayod County Case Data and FCM Evidence Base**

This supplement presents the complete dataset underlying the Ayod FCM analysis, including scenario definitions, the evidence repository, the baseline cognitive map, and full system state results with activation and predicted values.

**Dynamic FCM Scenario Definitions**

**Table S7: Dynamic FCM System Scenarios — Time Period and Description**

| **Dynamic FCM Scenarios** | **Period** | **Typical Livelihood Season** | **Events** |
| --- | --- | --- | --- |
| **System State 1** | July 2016 – November 2016 | Harvest period, livestock return to the homestead | Outbreak of national conflict and displacement into Ayod from Juba; atypical flooding in Ayod and additional internal displacement of populations in Ayod |
| **System State 2** | December 2016 – February 2017 | Dry season, some household food stocks remaining from harvest, livestock move away from the homestead | Outbreak of conflict in Ayod, with large advances into Ayod by SPLA, displacing populations in central Ayod to northern (Pagil) and western Ayod (low-lying Sudd) |
| **System State 3** | March to June 2017 | Late dry period and early rainfall. Lean season, a traditional period when household consumption patterns decrease | Conflict reduces slightly in Ayod; SPLA makes large advances in Canal/Pigi and Fangak counties, north of Ayod; cholera outbreak in Ayod; collapse of northern trade corridor—a critical food and livestock supply route into Ayod |
| **System State 4** | July to September 2017 | Early harvest, some green harvest available, livestock return to the homestead | Significant upscale in humanitarian food and nutrition distributions |

**Evidence Repository**

The evidence repository compiled for the dynamic FCM analysis included 242 unique entries categorized by date range, factor, source, and geographic location. This repository incorporated various data types, such as reports, assessments, datasets, and key informant interviews. It served as the foundational dataset for determining factor activation levels across scenarios.

**Table S8: Data Sources for Dynamic FCM Model Analysis**

| **Data Type** | **Source** | **Date** |
| --- | --- | --- |
| Annual Report | CMD Annual Report 2017 | 2017 |
| Assessment | REACH FSL Assessment / REACH FSL Data / REACH MUAC Screening | June – July 2017 |
| Assessment Report | WFP/CRS Rapid Needs Assessment | February – April 2017 |
| Focus Group Discussion Notes | SSD FGD Notes IDPs Ayod | April 2017 |
| Memo | Ayod Analysis Memo | August 2017 |
| Needs Assessment Report | Ayod County Inter-Agency Floods Needs Assessment | August – September 2016 |
| Population Movement Research | REACH Population Movement Baseline Research | 2017 |
| Project Proposal | Ayod SMART Project Proposal | August 2017 |
| Report | RMF SMART Survey Report | August – September 2017 |
| Report | FEWS NET Livelihood Zone Report | April 2017 |
| Report | Flooding Report CMD | September 2016 |
| Report | Jonglei FSL Report / REACH Canal Fangak FSL Report | April 2017 |
| Report | REACH FSL Report | June – July 2017 |
| Report | WFP Rapid Assessments / WFP RRM | January 2015 – September 2017 |
| Report | WFP – Planned HFA | October 2016 – May 2017 |
| Report | Conflict and Displacement Report IOM | December 2016 – January 2017 |
| Report | UNICEF SSD Situation Report | May 2017 |
| Report | WFP/UNICEF RRM Mission Normanyang | April 2017 |
| Report | Government of South Sudan Cholera Update | May – June 2017 |
| Report | IOM Situation Report | June 2017 |
| Report | IPC Worksheet / IPC May 2017 Update | May – September 2017 |
| Report | Jonglei Situation Overview | March 2017 |
| Report | REACH FGD – Nyal | September 2017 |
| Report | REACH FGD – Duk Padiet | March 2017 |
| Report | RMF Q1 2017 Project Report | January – March 2017 |
| Report | Acute Malnutrition and Jonglei Report by RMF | October – December 2016 |
| Report | Conflict Briefs (Jan – May 2017) | January – May 2017 |
| Report | UNICEF Situation Update | May 2017 |
| Report | UNICEF RRM Jan 2017 | January 2017 |
| Report | WHO Article – Cholera | July 11, 2017 |
| FGDs | REACH – Jonglei and Unity Area of Knowledge Bases | December 2016 – March 2017 |
| Research | REACH Population Movement Research | 2017 |
| Quantitative Data | WFP Actual HFA | April – September 2017 |
| Quantitative Data | CFSAM | August 2017 |
| Quantitative Data | FSNMS Round 20 | July 2017 |
| Quantitative Data | REACH Area of Knowledge FGDs | August 2017 |
| Quantitative Data | WFP RRM Data | October 2016 – September 2017 |
| Worksheet | IPC September 2017 Analysis Worksheet | September 2017 |

**Ayod Static (Baseline) Fuzzy Cognitive Map**

The baseline FCM model included 21 factors and 74 causal relationships, with a density of 0.176 and an average of 3.52 relationships per factor (21,24,25). The FCM’s complexity score was 0.25. Figure S3 provides a comprehensive visual of the famine system in Ayod and served as the basis for subsequent dynamic FCM analysis.

*Figure S3: Ayod Static (Baseline) Fuzzy Cognitive Map*

*Note: The Mental Modeler project file and FCM matrix table are available from the author upon request.*

**Results of System State FCM Analysis**

**Table S9: Results of System State FCM Analysis**

| **Factor** | **State 1 (Jul–Nov 2016)** | **State 2 (Dec 2016–Feb 2017)** | **State 3 (Mar–Jun 2017)** | **State 4 (Jul–Sep 2017)** |
| --- | --- | --- | --- | --- |
| Atypical Flooding | 1 | .75 | .5 | .75 |
| Livestock Access | –0.75 | –0.75 | –1 | –1 |
| Crop production | –0.5 | –0.75 | –1 | –0.5 |
| Market Functionality | –0.25 | –0.25 | –1 | –0.75 |
| Income Generation | –0.25 | –0.5 | –1 | –0.75 |
| Fishing Access | –0.5 | 0.25 | –0.75 | –0.5 |
| WASH | –0.5 | –0.5 | –1 | –1 |
| Cholera | 0.15 | 0.5 | 1 | 0.75 |
| IDPs | 0.25 | 0.75 | 1 | 0.5 |
| Disease Morbidity | 0.25 | 0.5 | 1 | 0.75 |
| Conflict – Internal | 0.25 | 1 | 0.5 | –0.25 |
| Conflict – External | 0.5 | 1 | 1 | 0.5 |
| HFA | –1 | –0.5 | –1 | 0.75 |
| Health and Nutrition Services | –0.25 | –0.5 | –1 | 0.25 |
| Outward Migration | 0.75 | –0.5 | –1 | –0.75 |
| Wild Food Consumption | 0.25 | 0.5 | 0.75 | 0.25 |
| Distress Migration | 0.15 | 0.5 | 1 | 0.75 |
| Caloric Intake* | –0.88 | –0.86 | –0.94 | –0.61 |
| Nutritional Status* | –0.46 | –0.6 | –0.65 | –0.17 |
| Health Status* | –0.38 | –0.56 | –0.61 | –0.25 |
| Mortality Risk* | 0.22 | 0.65 | 0.8 | 0.13 |

** Denotes FCM predicted values using dynamic FCM analysis*

**References**

21. Kosko B. Fuzzy cognitive maps. International Journal of Man-Machine Studies. 1986 Jan;24(1):65–75.

24. Gray SA, et al. Using fuzzy cognitive mapping as a participatory approach to analyze change, preferred states, and perceived resilience of social-ecological systems. E&S. 2015;20(2):art11.

25. Barbrook-Johnson P, Penn AS. Participatory Systems Mapping. In: Systems Mapping. Springer; 2022. p. 61–78.

**Supplement D: Validation Against Survey Data and Mortality Estimates**

This supplement presents the detailed validation of FCM predictions against empirical survey data and mortality estimates for Ayod County in 2017, together with a critical analysis of the methodological limitations of IPC assessments during the crisis.

**Household Food Security Survey Data**

The FCM analysis results were compared to outcomes from three separate surveys conducted between June and September 2017 to evaluate its predictive performance. Tables S10–S12 show the proportion of households from each survey and their indicative IPC Phase for household hunger score. Notably, FCM suggested famine-like conditions from March to June 2017, characterized by significant deterioration in caloric intake, disease prevalence, malnutrition, and mortality risk. This aligns with the findings of the REACH household survey, which reported that 29% of households had a Household Hunger Score (HHS) indicative of Phase 5 acute food insecurity (June 2017) (39).

FCM also predicted an improvement in conditions by August and September 2017, coinciding with the early harvest period and significant upscale in HFA. This is supported by FSNMS survey data, which reported 84% of households in Phase 3 based on HHS results (41). However, it is essential to note that the FSNMS data primarily covered southern and central Ayod, regions known for relatively better conditions. In contrast, the REACH survey included a broader geographic spread, covering northern, western, and central Ayod, providing a more comprehensive perspective.

**Table S10: REACH Household Survey Results (June 2017)**

| **Household Hunger Score** | **Percentage** | **n-count** |
| --- | --- | --- |
| Phase 3 | 41% | 33 |
| Phase 4 | 30% | 24 |
| Phase 5 | 29% | 23 |

**Table S11: FSNMS Household Survey Results (August 2017)**

| **Household Hunger Score** | **Percentage** | **n-count** |
| --- | --- | --- |
| IPC Phase 1 | 12% | 7 |
| IPC Phase 2 | 4% | 2 |
| IPC Phase 3 | 84% | 48 |

**Table S12: SMART Survey Results (August – September 2017)**

| **Household Hunger Score** | **Percentage** | **n-count** |
| --- | --- | --- |
| IPC Phase 2 | 8.40% | 36 |
| IPC Phase 3 | 82% | 354 |
| IPC Phase 4 | 9.60% | 41 |

**Nutrition Data**

The FCM effectively predicted shifts in malnutrition levels, aligning closely with survey data across various system states. During System State 2 (December 2016 – February 2017), FCM indicated a significant increase in malnutrition prevalence, corresponding with Global Acute Malnutrition (GAM) rates observed in early 2017, ranging from 26% to 34.2% (see Table S13).

In System State 3 (March – June 2017), FCM projected continued high malnutrition rates, supported by Mid-Upper Arm Circumference (MUAC)-based GAM results from screenings in Northern Ayod (34.2%) and the REACH survey (21.3%). Both results exceeded the Integrated Food Security Phase Classification (IPC) Phase 5 threshold of 15% GAM by MUAC (see Table S13).

System State 4 (July – August 2017) reflected FCM’s prediction of reduced yet elevated malnutrition levels. This matched SMART survey findings, which reported a GAM rate of 13.4% (see Table S13). However, FCM’s forecast suggested a slightly more optimistic decline than the survey data. Future refinements to the model could improve its accuracy in capturing the impact of humanitarian assistance and other external factors on malnutrition trends.

**Table S13: Timeline of Nutrition Screening and Surveys — Ayod County, 2017**

| **Date** | **Source** | **Methodology** | **Locations** | **Children Screened** | **GAM U5 (MUAC)** | **SAM U5 (MUAC)** |
| --- | --- | --- | --- | --- | --- | --- |
| Jan 12–16, 2017 | Kandak RRM | Mass MUAC Screening | Kandak (Northern Ayod) | 1,200 | 34.2% | 4.3% |
| Feb 1–4, 2017 | WFP-CRS RNA | Ad-Hoc Screening (Non-random) | Normanyang, Karmoun, Buot Wani | 115 | 26.0% | N/A |
| Apr 21, 2017 | Normanyang RRM | Mass MUAC Screening | Normanyang (Northern Ayod) | 1,713 | 34.2% | 8.17% |
| June 20–26, 2017 | REACH Household Survey | Two-stage PPS Random Sampling | Pagil, Gorwai, Jeich, Yein, Haat | 160 | 21.3% | 7.5% |
| Aug 21–Sept 16, 2017 | SMART Survey | Two-stage Cluster Sampling | Mongok, Pajiek, Wau, Pagil Payams | 596 | 13.4% | 4.5% |

*Note: GAM = Global Acute Malnutrition; SAM = Severe Acute Malnutrition; MUAC = Mid-Upper Arm Circumference; PLW = Pregnant and Lactating Women.*

**Comparison to Checchi et al. (2018) Mortality Model for South Sudan**

The FCM results were compared to a mortality model developed by Francesco Checchi et al. (2018). While Checchi’s model covers the period from December 2013 to April 2018 and does not distinguish between trauma and non-trauma mortality, it serves as a useful benchmark for assessing FCM’s ability to track relative mortality shifts (48).

Figure S4 presents normalized excess mortality estimates in Ayod County from July 2016 to September 2017, using a filtered subset of data from Checchi’s study (48). A Gaussian smoothing technique was applied to minimize short-term fluctuations, offering a clearer view of overall trends.

The graph shows a pronounced spike in normalized excess mortality from late 2016 into early 2017, followed by a further increase from May to September 2017 before a gradual decline into late 2017 and early 2018.

These trends partially align with FCM-predicted mortality risk values across the four periods:

• **July to November 2016:** 0.22

• **December 2016 – February 2017:** 0.65

• **March to June 2017:** 0.8

• **July to September 2017:** 0.13

While both the FCM predicted values and the mortality model suggest a significant increase in mortality in mid-2017, there are discrepancies in the timing of mortality reduction from System State 3 to System State 4. The Checchi et al. (2018) model indicates that the main spike in mortality rates occurred from July to September 2017, while the FCM suggests it happened between March and June 2017. These timing discrepancies warrant further investigation and refinement of contextual relationships to enhance predictive accuracy in future applications.

*Figure S4: Normalized Excess Deaths in Ayod County, South Sudan (July 2016 – September 2017)*

*Source: Francesco Checchi et al., 2018*

**Methodological Limitations of IPC Assessments — Ayod County, 2017**

A notable limitation of the IPC demonstrated by the Ayod case study is that rather than integrating AFI, AMN, and mortality data and previous IPC classifications into a cohesive system analysis, each survey was treated as a static observation (3,4,11). From January to September 2017, the IPC conducted three separate analyses of food insecurity and malnutrition in Ayod County. In January 2017, the IPC classified Ayod County as Emergency (IPC Phase 4) for both Acute Food Insecurity (AFI) and Acute Malnutrition (AMN) current period and throughout the projection period ending in September 2017 (38,41,65). In May 2017, an IPC Update analysis maintained AFI Phase 4 (Emergency) classification while AMN classifications worsened to IPC Phase 5 (38). This change was based on April screening data, particularly MUAC results showing proxy GAM rates above 30%, surpassing the Phase 5 threshold of 15%. In September 2017—with an analysis period of June to August—IPC TWG classified Ayod in IPC Phase 4 (Emergency) for both AFI and AMN.

The September 2017 IPC had access to multiple surveys conducted in the previous 90 days, each with distinct limitations.

• **Household Survey 1 (REACH, June 2017):** Reported Phase 5-level AFI with adequate geographic coverage and sample size. AMN results suggested Phase 4/5, though based on a small sample.

• **Household Survey 2 (FSNMS, July–August 2017):** Indicated AFI at Phase 4/5, though the sample size and geographic scope were limited. No AMN or mortality data were collected.

• **Household Survey 3 (SMART, August–September 2017):** Reported AFI and AMN outcomes at Phase 3/4. More than 50% of surveyed clusters had received humanitarian food assistance (HFA) within two weeks before data collection, introducing potential response-related bias. Mortality data estimated a crude death rate (CDR) of 1.89 (95% CI: 1.26–2.84) per 10,000 people per day, covering a recall period from May 16 to September 2017. Over half of reported deaths occurred during migration, indicating clustering in displacement settings. A design effect of 3.52 further suggests substantial mortality clustering.

In the initial presentation of the SMART survey results, the non-trauma CDR was 2.03, exceeding the Phase 5 threshold for mortality (49). However, the CDR was later reduced to 1.89 in the final report (50). Comparing the two documents, the decrease stems from an extension of the recall period from 107 to 126 days. According to a final presentation of the findings, the recall period was extended due to weather-related cancellation of UNHAS flights delaying data collection in the final clusters. However, no additional deaths were captured in the delayed clusters, yet the expanded recall period resulted in the preliminary non-trauma CDR in Ayod from May 2017 to August 2017 decreasing below the famine threshold (2 deaths per 10,000 people per day).

**References**

3. Maxwell D, Hailey P. Towards Anticipatory Information Systems and Action: Notes on Early Warning and Early Action in East Africa. Boston: Feinstein International Center, Tufts University; 2020.

4. Maxwell D, Khalif A, Hailey P, Checchi F. Viewpoint: Determining famine: Multi-dimensional analysis for the twenty-first century. Food Policy. 2020 Apr;92:101832.

11. Maxwell D, Hailey P. The Politics of Information and Analysis in Famines and Extreme Emergencies: Synthesis of Findings from Six Case Studies. Boston: Feinstein International Center, Tufts University; 2020.

48. Checchi F, Testa A, Warsame A, Quach L, Burns R. Estimates of crisis-attributable mortality in South Sudan, December 2013–April 2018. London School of Hygiene and Tropical Medicine; 2018.

38. Integrated Food Security Phase Classification (IPC). South Sudan: Acute Food Insecurity Situation for May 2017 and Projection for June–July 2017. 2017.

39. REACH Initiative. Ayod Rapid Assessment Report. 2017.

41. Integrated Food Security Phase Classification (IPC). South Sudan: Acute Food Insecurity Situation September 2017 and Projection for October–December 2017. 2017.

50. Real Medicine Foundation. SMART Survey Final Report Ayod, South Sudan. 2017.

49. Real Medicine Foundation. Ayod County, Jonglei State Preliminary nutrition SMART survey findings. 2017.

65. South Sudan: Acute Food Insecurity Situation for January 2017 and Projections for February–April 2017 and May–July 2017. IPC.

**Supplement E: FCM System Dynamics, Feedback Loops, and Assessment**

This supplement presents the extended systems analysis from the Ayod FCM, including the identification of tipping points, emergent properties, and self-reinforcing dynamics that static classification methods do not capture, together with a detailed assessment of FCM’s strengths and weaknesses.

**Value Add of FCM for Analyzing and Understanding Famine Systems**

Developing a baseline FCM provided the author with a tool to visualize and analyze the complex interrelationships influencing Ayod’s trajectory toward famine. The baseline FCM served as both an analytical framework and tool. The resultant map supported identifying critical linkages and feedback loops driving the system’s trajectory, offering insights into the various traits described by Howe (1) and Fortnam and Hailey (2). In summary, a contextualized famine system blueprint. For Ayod, the FCM demonstrated how flooding’s initial impact on food production and livestock access (System State 1) was exacerbated by conflict and deteriorating WASH conditions (System State 2). By System State 3, self-reinforcing dynamics were evident, potentially driving the system toward food system collapse, distress migration, and cholera spread—a possible tipping point (25).

Figure S5 highlights this tipping point, where prolonged pressures (downward arrows) and collapsing adaptive capacity (upward arrows) accelerated feedback loops (diagonal arrow), contributing to systemic destabilization and heightened famine risk. During this state, the FCM indicated that converging factors—malnutrition, disease outbreaks, and food scarcity—could lead to rapid declines in caloric intake, nutritional status, and health. Mortality risk was projected to increase by approximately 0.8.

*Figure S5: Ayod Famine System Trajectory*

The model also demonstrated how humanitarian food assistance (HFA) acted as a rebalancing mechanism in System State 4, improving adaptive capacity and disrupting self-reinforcing dynamics. The rise in mitigating factors gradually alleviated famine conditions, steering the system away from a famine state. By modeling these interactions, FCM provided a clearer understanding of when and how famine conditions could emerge, enhancing early warning capabilities and supporting intervention timing.

**Capturing Nonlinear and Emergent Properties**

FCM supported identification and characterization of nonlinear interactions and emergent properties in Ayod during 2016–17. Nonlinear dynamics became pronounced between February and July 2017, driven by self-reinforcing feedback loops that rapidly escalated famine conditions. Severe food shortages and depletion of household reserves triggered distress migration within Ayod, exacerbating public health crises, particularly cholera, as populations concentrated in unsanitary environments such as cattle camps and stagnant water sites.

*Emergent Properties Driving Ayod toward Famine*

Three primary factors illustrate the emergent properties contributing to famine conditions in Ayod:

• **Atypical Flooding (August–October 2016):** Severe flooding caused substantial crop and livestock losses, depleted household assets, and accelerated the onset of the lean season, significantly worsening food insecurity.

• **Internal and External Conflict:** Internal conflict (January–March 2017) displaced populations, while external hostilities impeded access to markets, humanitarian aid, and health services, forcing households to increasingly rely on wild foods.

• Cholera Outbreak: Initiated by conflict-induced displacement in July 2016, cholera rapidly spread throughout South Sudan. By February 2017, reliance on contaminated water and worsening food security resulted in over 3,000 probable cholera cases in March 2017—the highest concentration of probable cases in four years (47,51).

These interconnected factors amplified each other’s impact: flooding heightened cholera transmission risks, and conflict limited humanitarian interventions. FCM’s systemic analysis clarified these interactions, revealing how combined shocks from flooding, conflict, and disease intensified famine conditions.

**Self-Reinforcing Dynamics and Feedback Loops**

The Ayod famine system featured prominent self-reinforcing dynamics and interconnected feedback loops. Initial shocks, including severe food production and livestock losses, forced increased reliance on wild foods, resulting in malnutrition and illness. Flooding and escalating conflict caused significant internal displacement into northern and western Ayod, accelerating resource depletion and compelling populations to travel further in search of wild foods. Increased intra-Ayod displacement in search of wild foods amplified cholera transmission, markedly intensifying health and nutritional crises from March to September 2017. Concurrently, heightened conflict around Ayod severely restricted outward mobility, limiting the system’s adaptive capacity. Reduced mobility compounded local resource pressures, disease transmission, and competition for scarce resources, culminating in widespread mortality. Ultimately, systemic equilibrium emerged primarily through mortality, illustrating system collapse driven by entrenched feedback loops involving resource depletion, restricted mobility, disease prevalence, and escalating malnutrition. Figure S6 highlights key factors and critical relationships underpinning these self-reinforcing dynamics from the original baseline FCM presented in Figure S3 (Supplement C).

*Figure S6: Notable Factors and Relationships*

**Strengths and Weaknesses of FCM in the Ayod Case**

There are multiple limitations that should be considered when interpreting these findings. First, it represents a single application of FCM to one famine context. While the Ayod case study demonstrates FCM’s potential utility, broader conclusions about its general applicability require further testing across diverse settings. Second, the Ayod FCM was developed by the author using available evidence and expertise, rather than through a participatory process involving a range of stakeholders. Future applications should prioritize participatory development, incorporating the perspectives of affected communities and other relevant actors. Third, validating FCM outputs against empirical outcomes remains difficult in humanitarian contexts with limited data. Further research is needed to establish rigorous validation protocols for FCM in famine analysis and early warning.

**Table S14a: Strengths of FCM in the Ayod Case**

| **Strengths** | **Description** |
| --- | --- |
| **Flexibility and Adaptability** | Integrates diverse data types (qualitative, quantitative, expert judgment), supporting analysis in imperfect information environments. |
| **Scenario Analysis Capability** | Allows assumption tracking and simulation of system behavior under alternative conditions. Supports early warning and adaptive planning. |
| **Integration of Diverse Perspectives** | Incorporates local knowledge, field observations, and expert opinion, enhancing contextual grounding. |
| **Real-Time Monitoring Blueprint** | Identifies critical factors and relationships for RTM systems; supports prioritization of data collection. |
| **Decision-Making Utility** | Helps identify leverage points; structures interpretation of system dynamics relevant to outcomes like malnutrition or mortality. |

**Table S14b: Weaknesses of FCM in the Ayod Case**

| **Weaknesses** | **Description** |
| --- | --- |
| **Subjectivity and Bias** | Relationship weights and factor selection rely on expert judgment, which can introduce inconsistencies or biases. |
| **Simplification of Complex Relationships** | FCM necessarily simplifies real-world dynamics, potentially oversimplifying time-delayed effects, non-linear interactions, or threshold effects in the famine system. |
| **Scalability and Complexity** | Large, highly connected models can become difficult to interpret and update, particularly in multi-sectoral crises. Cognitive limitations affect our ability to conceptualize highly complex systems. |
| **Communication to Non-Experts** | Technical outputs may be difficult to convey to decision-makers without strong visual or narrative translation. |
| **Limited Integration with Other Methods** | Aligning FCM outputs with other SATs (e.g., ACH, household-level analysis) requires additional design and coordination. |
| **Dependence on Iterative Updating** | Requires sustained engagement and regular updates to remain relevant; otherwise, risks becoming static or outdated. |
| **Standardization Challenges** | Unlike IPC’s standardized protocols, FCM applications can vary considerably in design and implementation, complicating quality control and cross-context comparison. |

**References**

1. Howe P. Famine systems: A new model for understanding the development of famines. World Development. 2018 May;105:144–55.

2. Fortnam M, Hailey P. Insights from social-ecological systems thinking for understanding and preventing famine. Disasters. 2024 Jul;48(3):e12621.

25. Barbrook-Johnson P, Penn AS. Participatory Systems Mapping. In: Systems Mapping. Springer; 2022. p. 61–78.

51. Government of South Sudan, World Health Organization. South Sudan Cholera Outbreak Update. 2017.

47. Jones FK, et al. Successive epidemic waves of cholera in South Sudan between 2014 and 2017. The Lancet Planetary Health. 2020 Dec;4(13):e577–87.
